# A Comparison of Multiscale Permutation Entropy Measures in On-Line Depth of Anesthesia Monitoring

**DOI:** 10.1371/journal.pone.0164104

**Published:** 2016-10-10

**Authors:** Cui Su, Zhenhu Liang, Xiaoli Li, Duan Li, Yongwang Li, Mauro Ursino

**Affiliations:** 1 Department of Electrical Engineering, Yanshan University, Qinhuangdao, 066004, China; 2 Department of Information Science and Engineering, Yanshan University, Qinhuangdao, 066004, China; 3 Department of Anesthesiology, The Second Artillery General Hospital, Beijing, 100088, China; 4 Department of Electrical, Electronic and Information Engineering, University of Bologna, Viale Risorgimento 2, Bologna, l40136, Italy; Universidad Veracruzana, MEXICO

## Abstract

**Objective:**

Multiscale permutation entropy (MSPE) is becoming an interesting tool to explore neurophysiological mechanisms in recent years. In this study, six MSPE measures were proposed for on-line depth of anesthesia (DoA) monitoring to quantify the anesthetic effect on the real-time EEG recordings. The performance of these measures in describing the transient characters of simulated neural populations and clinical anesthesia EEG were evaluated and compared.

**Methods:**

Six MSPE algorithms—derived from Shannon permutation entropy (SPE), Renyi permutation entropy (RPE) and Tsallis permutation entropy (TPE) combined with the decomposition procedures of coarse-graining (CG) method and moving average (MA) analysis—were studied. A thalamo-cortical neural mass model (TCNMM) was used to generate noise-free EEG under anesthesia to quantitatively assess the robustness of each MSPE measure against noise. Then, the clinical anesthesia EEG recordings from 20 patients were analyzed with these measures. To validate their effectiveness, the ability of six measures were compared in terms of tracking the dynamical changes in EEG data and the performance in state discrimination. The Pearson correlation coefficient (*R*) was used to assess the relationship among MSPE measures.

**Results:**

CG-based MSPEs failed in on-line DoA monitoring at multiscale analysis. In on-line EEG analysis, the MA-based MSPE measures at 5 decomposed scales could track the transient changes of EEG recordings and statistically distinguish the awake state, unconsciousness and recovery of consciousness (RoC) state significantly. Compared to single-scale SPE and RPE, MSPEs had better anti-noise ability and MA-RPE at scale 5 performed best in this aspect. MA-TPE outperformed other measures with faster tracking speed of the loss of unconsciousness.

**Conclusions:**

MA-based multiscale permutation entropies have the potential for on-line anesthesia EEG analysis with its simple computation and sensitivity to drug effect changes. CG-based multiscale permutation entropies may fail to describe the characteristics of EEG at high decomposition scales.

## 1. Introduction

Since biological systems are highly integrated systems functioning at multiple time scales, biosignals often exhibit the characteristics of multiple scales [1]. Multiscale entropy (MSE) analysis has been developed to detect the dynamic changes of multiscaled signals to analyze the correlations of time series over multiple temporal scales, which could offer extra information than single scale [2–6].

To describe the multiscale property of neural signals, many attempts have been made to develop MSE algorithms. Typically, Costa et al. proposed a MSE based on the consecutive coarse-graining (CG) procedure combined with approximate entropy (AE) [7] to assess the complexity of time series [2, 4, 8, 9]. Li et al proposed the multiscale permutation entropy (MSPE) based on CG to track the effect of sevoflurane anesthesia on the central nervous system. The results showed that MSPE measures process the capability to describe the subtle transition from light anesthesia to deep anesthesia accurately, whereas sing-scale permutation entropy cannot distinguish the two states apparently [10]. Meanwhile, MSE based on CG procedure has been used to analyze the dynamic of physiological time series in many other studies as well [5, 10–12]. However, this coarse-graining process reduces the length of a time series as the decomposition scale increases. When applied to a short-term time series in high decomposition scale, it may yield an imprecise entropy index [13]. To overcome this shortcoming, Wu proposed the moving-averaging (MA) procedure to reduce the decomposition impact on the data length [13] and evaluated the effectiveness of CG and MA by synthetic noise signal analysis.

However, the existing MSE methods are designed for off-line analysis. There is a lack of on-line MSE methods to provide real-time depth of anesthesia (DoA) information during surgery. At present, there are several commercialized DoA monitors used in clinic, such as the bispectral index, entropy module, Nacrotrend, etc [14]. These methods are based on frequency domain information and analyze EEG signal on single scale, thus they cannot provide comprehensive information from narcosis patients as MSE methods can. So there will be potential risks in estimating the state of patients with these indexes in general anesthesia. It is of great significance to investigate the use of on-line MSE methods for analyzing EEG data during surgery.

In terms of entropy applied in MSE, Shannon entropy (ShEn) [15], sample entropy (SampEn) [16], permutation entropy (PE) [17] and some other entropies are usually considered. Actually, permutation entropy measures have been proven to have a better performance in anesthesia EEG analysis [10, 18]. Especially, Pil-Jong Kim et al. found that PE seem to be a useful indictor of DoA in children and have a comparable performance to BIS index [19]. Notably, the classic definition of PE is based on Shannon information theory, which is a short-range and extensive concept [18]. Considering that the neural system usually processes in long term over multiscale, to tackle this issue, two generalized forms of permutation entropy were proposed: Renyi permutation entropy (RPE) [18, 20] and Tsallis permutation entropy (TPE) [21]. In our previous study, it has been proved that three PE measures (Shannon PE (SPE), RPE and TPE) outperform the other entropy indexes and RPE has the best performance [18]. Herein, the SPE, RPE and TPE were chosen for MSE measures construction.

In this paper, we combined two decomposition methods CG and MA with SPE, RPE and TPE measures to construct six MSPE methods, i.e. CG-SPE, CG-RPE, CG-TPE, MA-SPE, MA-RPE and MA-TPE. In previous studies, MSEs are designed for data analysis after data collection, while, in this paper the MSPEs were tested for on-line data analysis to find a possible index indicating the depth of anesthesia during surgery. The on-line EEG recorded from patients was processed with moving-window method before MSPE analysis, thus the required data length is much smaller than off-line analysis.

To validate the effectiveness of the proposed methods, a thalamo-cortical neural mass model (TCNMM) based on neurophysiological mechanisms is adapted to generate neural populations and simulate anesthesia EEG signal [22]. The model can be used to simulate the neural signal to discover the mechanisms of brain activity and the relationship between different brain regions and it has been used to simulate brain rhythms during sleep in [22]. Anesthesia is characterized with unconsciousness, decrease in global cerebral metabolism [23] and high-voltage low frequency EEG [24, 25], which are also the characteristic features of non-rapid eye movement (NREM) sleep. Especially, both propofol anesthesia and NREM sleep can cause spindles in EEG [26, 27]. The apparent similarities of anesthesia and sleep in both behaviors and EEG signals show the possibility that the model could simulate anesthesia signals. It should be noted that the EEG signal produced by this model is pure signal without noise, it can be utilized to assess the anti-noise capability of different EEG processing methods by adding different intensity of noise to the produced noise-free signals. The produced signal offers an effective test platform for EEG signal processing methods before applied to the clinical analysis.

In this paper, the thalamo cortical neural mass model was introduced to generate noise-free EEG data consisting awake state and unconscious state. Then, white noises of different intensities were added to the generated signals to evaluate the anti-noise ability of MSPE methods. The MSPE measures were evaluated with clinical EEG in quantifying the anesthetic drug effect as well. Further, correlation analysis between all the MSPEs was computed to assess their relationship.

## 2. Methods

MSPE measures were used to detect the dynamic changes of on-line anesthesia EEG. In this paper, six MSPEs were constituted by three permutation entropies, i.e. SPE, RPE and TPE merged with two decomposition methods CG and MA.

### 2.1 Multiscale decomposition methods

#### 2.1.1 Coarse-graining procedure

**Fig 1:**
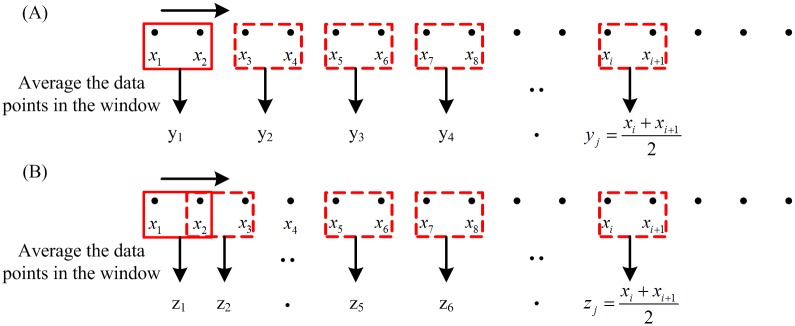
Illustrations of multiscale decomposition procedures. (a) Coarse-graining procedure for scale = 2. The window size is the scale level s. (b) Moving average procedure for scale = 2. The window size is the scale level s, and overlap size is s − 1.

Given a one-dimensional discrete time series {*x*_1_, *x*_2_, …*x*_*i*_, …, *x*_*N*_}, construct a set of consecutive coarse-grained time series {*y*^(*s*)^}, where *s* is the scale factor. As shown in Fig 1(A), each coarse-grained time series is obtained according to the following equation,

$$y^{\left(s\right)}=\frac{1}{s}\sum_{i=\left(j-1\right)s+1}^{js}x_{i},\;1\leq \text{j}\leq \text{N}/\text{s}$$ (1)

The length of time series processed by coarse-graining equals to N/s, where N is the length of the original time series and s is the decomposition scale [10].

#### 2.1.2 Moving average procedure

Similar to the coarse-graining procedure, each element of a moving average time series is defined as follows [13],

$$z_{j}^{\left(s\right)}=\frac{1}{s}\sum_{i=j}^{j+s-1}x_{i},\;1\leq \text{j}\leq \text{N}-\text{s}+1$$ (2)

The detail of moving average procedure is shown in Fig 1(B). Compared to the CG procedure, the moving average procedure has little impact on the length of the new time series. The length of the moving-averaged time series is (N−s+1). And this makes moving average procedure more reliable than CG procedure for short-term time series analysis.

### 2.2 Permutation entropy

Permutation entropy was originally proposed by Bandt and Pompe [17, 28]. It reveals the order information of signals by reconstructing the given time series into ordinal patterns. It has been used to analyze neural signals successfully [29–31]. There are three types of PE measures considered in this study, including SPE, RPE and TPE.

Suppose the length of the given signal is K. Divide the signal into several vectors consisting of N consecutive data taken from the signal by moving-window method. Express the new vector as {x(i): 1 ≤ i ≤ N}. First, reconstruct the vector into *X*_*t*_ = [*x*_*t*_, *x*_*t*+*τ*_, ⋯, *x*_*t*+(m−1)*τ*_] with the embedding dimension m and lag τ. Then, rearrange *X*_*t*_ in an increasing order. The vector *X*_*t*_ consists of m different values, so there will be m! possible patterns *π*_*i*_, which is also known as permutations. Each vector necessarily belongs to 1 of m! possible patterns. Adopt a symbolic representation on the basis of the data level within the vector. For each pattern *π*_*i*_, *f*(*π*_*i*_) denotes its frequency of occurrence in the vector. The relative frequency is $\text{p}\left(\pi_{i}\right)=\frac{f(\pi_{i})}{N-(m-1)\tau }$, where *N* is the length of the vector. The normalized Shannon permutation entropy is defined as

$$\text{SPE}=\frac{-\sum_{i=1}^{m!}\text{p}\left(\pi_{i}\right)ln\text{p}\left(\pi_{i}\right)}{\text{ln}(m!)}$$ (3)

Based on the Renyi entropy and permutation probability distribution p(*π*_*i*_), the normalized RPE measure is proposed and defined as:

$${\text{RPE}}_{n}=\frac{log\sum_{j=1}^{m!}\text{p}{\left(\pi_{i}\right)}^{a}}{\left(1-a\right)*\text{ln}\left(m!\right)}$$ (4)

Zunino et al proposed the normalized TPE based on the definition of Tsallis entropy [32]:

$${\text{TPE}}_{n}=\frac{1}{1-m{!}^{1-q}}\Sigma_{j-1}^{m!}(\text{p}\left(\pi_{i}\right)-\text{p}{\left(\pi_{i}\right)}^{q})$$ (5)

In this way, each vector is given a symbolic value from 0 to 1, which represents the ordering information of the vector. Therefore, the normalized PEs range from 0 to 1. The maximum value is 1, which means that all patterns have equal probability. The smallest value is 0, which implies that the time series are extremely regular. The value of SPE is reversely proportional to the regularity level of the time series [10].

Obviously, m, τ and N are the main parameters in SPE computation. The dimension m must satisfy (m)! < N. However, if m is too small, there are very few patterns and the computation will be nonsense. And if m is too large, the computation of the phase space reconstruction will also grow exponentially. For the time delay τ, it is adequate to select a common value of τ = 1 to extract most of the information in the EEG [29, 33, 34]. The details of the parameters selection have already been discussed before [18]. Since the EEG dataset was different with previous study in this paper, the selection of computation parameters were discussed in the appendix. It is suggested that m = 6 and τ = 1 are suitable for SPE, m = 6, τ = 1 and a = 2 are selected for RPE, m = 6, τ = 1 and q = 0.1 have a better performance in TPE calculation.

### 2.3 Multiscale permutation entropy measures

Six MSPE measures, namely: CG-SPE, CG-RPE, CG-TPE, MA-SPE, MA-RPE, MA-TPE, were formed by means of combining three PE measures with two multiscale decomposition methods, respectively. In terms of the decomposition level, five decomposition scales from 1 to 5 were chosen. For scale 1, the MSPEs are simply the original PEs. The MSPEs were compared with PEs to verify their effectiveness.

In this paper, the MSPEs were designed for on-line DoA monitoring. The data was processed with moving-window method before MSPE analysis. The calculation depends on the length of window N and the embedding dimension *m*. It should be noted that there is a necessary condition (m)! < N [34]. According to the introduction in 2.2, if m is set to 6, the length of chosen epoch needs to be bigger than 720 at each scale. The length of time series analyzed by MA just has a little change and makes no difference in the epoch length, while the length of time series analyzed by CG is N/s, which violates the rule (m)! < N at *N* = 1000 in case of *s* ≥ 2. This requirement can be solved by two ways: extending *N* and decreasing m to the second-best option m = 3. For CG-based MSPEs, the selection process of N and m will be discussed with the simulated EEG signals in the next section.

## 3. Simulation and Results

### 3.1 Thalamo-cortical neural mass model

In this paper, a TCNMM [24, 25] consisting of two thalamic populations and four cortical populations was introduced. The two thalamic populations are thalamo-cortical relay cell population (TCR) and thalamic reticular nucleus population (TRN). The cortical populations are made up of pyramidal neurons, excitatory interneurons, inhibitory interneurons with slow and fast kinetics. The model helps understand the dynamic characteristics under different physiological or pathological conditions. The construction of the model is presented in the appendix, while more details can be found in [22]. As the produced EEG signal is noise free, it could be utilized to test the anti-noise ability of the six measures and select the appropriate computation parameters of MSPEs.

This model has been used for the simulation of brain rhythms during sleep [22]. In this paper, it is utilized to mimic the progressive changes between awake, anesthesia and RoC (Recovery of Consciousness) states, by choosing proper modulatory inputs. This is done keeping in mind that sleep and anesthesia are both characterized by the loss of consciousness, behavioral immobility and little recall of environmental events [35]. And it has been verified that the anesthetic effect may also be mediated through the brain nuclei that control sleep-wake states [36, 37]. Especially for the GABAergic (GABA = gamma-amino-butyric acid) anesthetic drugs, the EEG effect shows regular oscillation changes with the deepening of anesthesia. The anesthetics change characteristics of the EEG signal from high frequency-low amplitude to low frequency-high amplitude, and these waves are related to the anesthetic drug concentrations. First, during normal resting stages the spectral distribution of the EEG shows a strong suppression of alpha and beta power bands, and a dominance of slow wave delta/theta power bands [38]. Then, the EEG power in the high-frequency range is decreased, and the EEG signals mainly lie in theta and delta power bands as the anesthetic concentration increases. Finally, deep anesthesia may rise to the burst suppression pattern [39].

To produce the anesthesia EEG data, three modulatory inputs, namely inputs reaching the TCR (*I*_*M*,*T*_), the TRN (*I*_*M*,*R*_) and the pyramidal (*I*_*M*,*P*_) populations, are modulated to switch between different states. The modulations are provided to make sure the power spectrum of the produced signal meets the power spectrum of clinical EEG in different states.

### 3.2 Results

**Fig 2:**
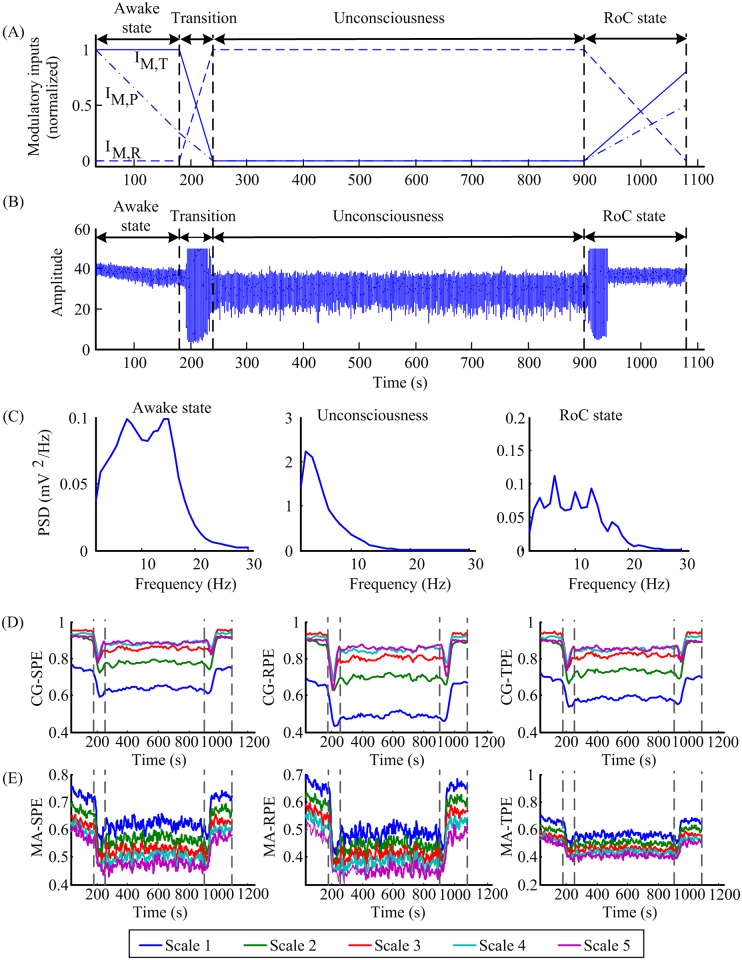
Simulated EEG signal generated by tuning three modulatory inputs and the corresponding MSPE indexes. (A) Three normalized modulatory inputs changed with time. I_*M*,*T*_, I_*M*,*R*_ and I_*M*,*P*_ represent the inputs to TCR, TRN and pyramidal cells, respectively. (B) The simulated EEG signal obtained from the TCNMM model. (C) The power spectral densities of the signal in different anesthesia states. (D) The CG-SPE, CG-RPE and CG-TPE indexes computed from the generated signal. (E) The MA-SPE, MA-RPE, MA-TPE indexes computed from the generated signal. In (D-E), the MSPE indexes were delimited by vertical bars into four states: awake state, transition, unconsciousness and RoC state. *I*_*M*,*T*_, *I*_*M*,*R*_ and *I*_*M*,*P*_ represent modulatory inputs for the TCR, TRN and the pyramidal population, respectively.

To mimic the gradual transition during anesthesia, three modulatory inputs were turned progressively as displayed in the Fig 2(A) (normalized) and the values in different states were summarized in Table 1. The process is divided into four phases: awake state, transition, unconsciousness and RoC state. The average membrane potential of pyramidal neurons, i.e. the approximation of cortical EEG, is shown in Fig 2(B). Fig 2(C) shows the power spectral densities during each phase that corresponds to the general EEG features during clinical anesthesia [24, 25]. The patients had normal and active EEG before anesthesia. With the deepening of anesthesia, there was a decrease in beta activity and an increase in theta and delta activity. During the RoC state, the EEG patterns acted in approximately reverse order form anesthesia to awake state [40].

**Table 1:** Modulatory inputs for TCNMM.

	Awake state	Unconsciousness	RoC state
*I*_*M*,*T*_	4.5	2	4
*I*_*M*,*R*_	-5.5	-5	-5.5
*I*_*M*,*P*_	130	50	90

**Fig 3:**
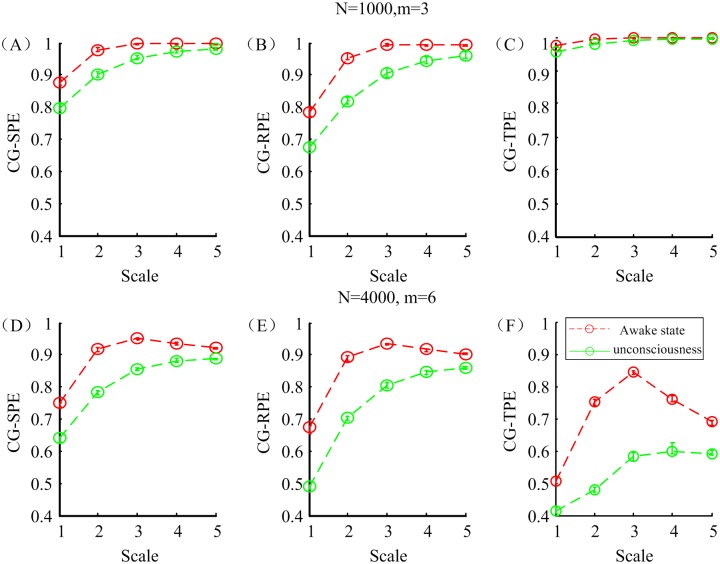
The CG-SPE, CG-RPE and CG-TPE indexes with different parameters in awake and anesthesia states. (A-C) The mean and standard deviation of three indexes with parameters: N = 1000, m = 6, τ = 1, a = 2 and q = 0.1 (D-F) The mean and standard deviation of three indexes with parameters: N = 4000, m = 6, τ = 1, a = 2 and q = 0.1.

Parameter selection of CG-based MSPEs computation was carried out to find proper parameters. Two sets of EEG data, referring to awake state and unconsciousness, were picked up from the simulated EEG and down sampled to 100 Hz. The selection criteria was based on the performance in distinguishing awake and anesthetic states. Two parameter groups N = 1000, m = 3 and N = 4000, m = 6 were designed for comparison. Other parameters were set as τ = 1, a = 2 and q = 0.1 according to the selection result in the appendix [18]. Fig 3 shows the changes of CG-SPE, CG-RPE and CG-TPE at five scales based on two sets of parameters in different states. All the indexes monotonously decreased in anesthesia state. It is obvious that in the N = 4000, m = 6 group, the differences between two states were more significant and this indicated that the second group of parameters had better ability in distinguishing awake and anesthesia state. Therefore, N = 4000, m = 6, τ = 1, a = 2 and q = 0.1 were selected for CG-based MSPEs and N = 1000, m = 6, τ = 1, a = 2 and q = 0.1 for MA-based MSPE.

**Fig 4:**
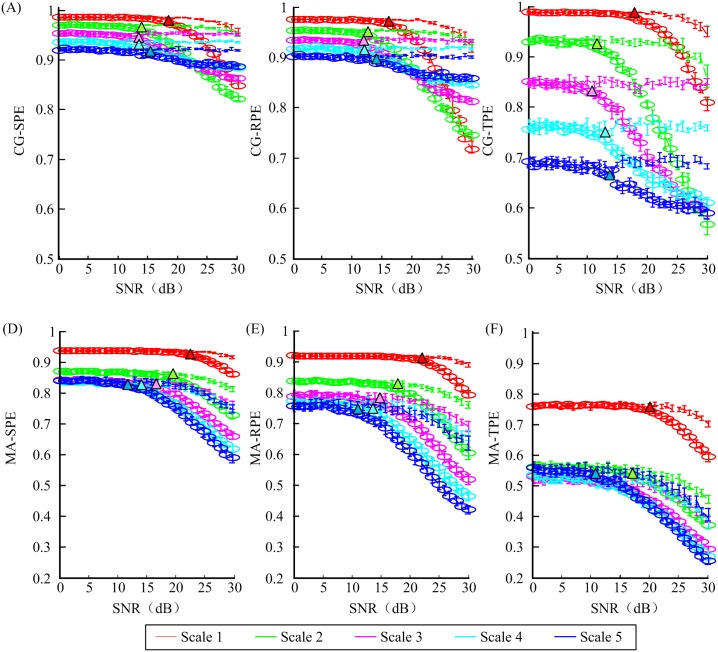
The values of six MSPEs versus signal to noise rate (SNR) in awake state (lines without circle) and unconsciousness (lines with circle). Different colors represent different scales. Colored triangle symbols pointed out the SNR value where the indexes could tell awake state and anesthesia state apart.

In order to assess the robustness of each entropy measure against noise, white Gaussian noise with different intensities were added to the selected signal used in the parameter selection part. The noise level was set so that SNR linearly increases from 0 to 30 dB. As shown in Fig 4, six MSPE measures were applied to these 31 sets of noise-added signals with a moving-window technique [41]. The SNR thresholds, below which MSPEs cannot separate awake state and unconsciousness, are summarized in Table 2. Lower SNR threshold represents better anti-noise ability.

**Table 2:** The thresholds of SNR.

	Scale 1	Scale 2	Scale 3	Scale 4	Scale 5
CG-SPE	15	11	10	10	13
CG-RPE	14	11	10	10	13
CG-TPE	16	11	10	13	14
MA-SPE	21	18	16	13	10
MA-RPE	19	17	12	11	9
MA-TPE	20	18	16	15	11

Overall, CG-based MSPE measures had lower SNR thresholds indicating better anti-noise ability than MA-based MSPEs. The thresholds decreased with the increase of scales, except CG-based measures at scale = 4,5. Compared to single-scale PEs, higher decomposition scale can increase the robustness against white Gaussian noise. CG-based measures at scale = 4,5 clashed with the conclusion, since the length of the signal had been shortened too much, leading to signal distortion. In the horizontal comparison, the results showed that RPE-based MSPEs outperformed others in anti-noise ability with smallest SNR threshold.

Taking advantage of the selected parameters, the corresponding MSPEs of the produced anesthesia signal were computed and shown in Fig 2(D) and 2(E). It was obvious that all the indexes decreased in the unconsciousness and increased during the RoC state, which corresponds to the assumption that the MSPE values in the awake state will be maximum, minimum in the unconsciousness. It verified that the produced signal generated by the TCNMM model could be used for MSPEs computation and MSPEs could track the dynamic features of anesthesia EEG signal.

## 4. Application to Clinical Anesthesia EEG Recording

### 4.1 EEG data recording and preprocessing

In this study, the EEG recordings were obtained from 20 patients aged from 20 to 65, who were classified as American Society of Anesthesiologists (ASA) physical status I or II. No special etiologies and detectable underlying structural abnormality had been found. Written informed consents were obtained for each participant according to the study protocol approved by the ethics committee of second artillery general hospital of Chinese people’s liberation army.

Before surgery, patients were given midazolam and sufentainil for sedation. Then midazolam, sufentainil, remifentanil and cisatracurium were injected for anesthesia induce. During the surgery, remifentanil, propofol and dexmedetomidine hydrochloride injection were adjusted to maintain anesthesia.

The EEG data was recorded by the Bio-Acquisition Systems (Bio-AMP8, Kangpu Medical, Huzhou, Zhejiang) and consisted of pre-operation, operation and RoC state. The electrodes were placed at the position of Fpz, Fp1 and F8 according to the 10–20 international standard system and F8 was the ground electrode.

The sampling rate of EEG recording was 1000 Hz. First the low frequency baseline drift and head movement noise were removed. Then, the data points with the absolute amplitude values exceeding 300 μV were removed as the outliers. The signals were further preprocessed by a statistical threshold, which was set as mean±2SD. Then the main 50 Hz linear noise was removed by a classical adaptive notch canceling method. Inverse filtering was used to remove EMG and other high-amplitude transient artifacts [42]. The filter was adjusted by the least-mean-square adaptive algorithm [43].

### 4.2 Results

**Fig 5:**
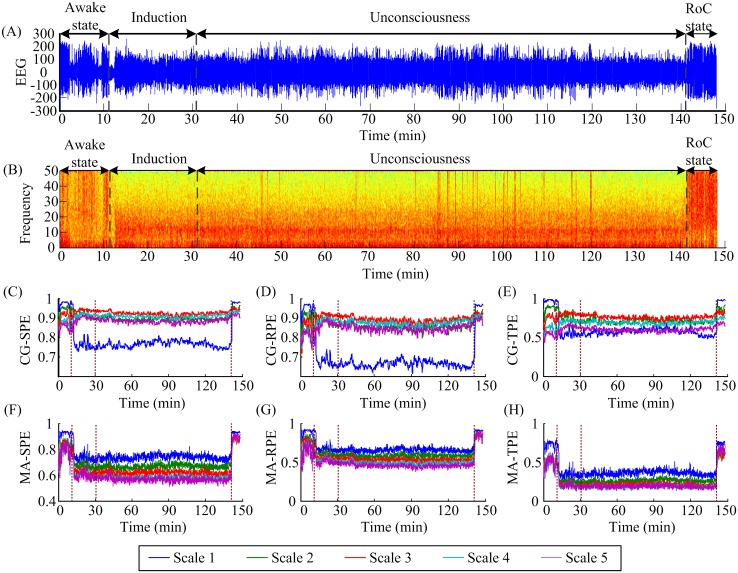
An EEG recording from one patient, its spectrogram and corresponding MSPE indexes. (A) The EEG recording. The EEG recordings are divided into four states: awake state, induction, unconsciousness and RoC states. (B) The spectrogram of the EEG signal. (C-H) The corresponding CG-SPE, CG-RPE, CG-TPE, MA-SPE, MA-RPE and MA-TPE indexes of the signal at five decomposition scales. Different colors represent different decomposition scales.

First six MSPE measurements (CG-SPE, CG-RPE, CG-TPE, MA-SPE, MA-RPE, MA-TPE) at five scales were calculated on anesthesia EEG signals recorded from 20 patients. CG-based measures were computed over a window of 40 s with an overlap of 20 s and MA-based measures were computed over a window of 10 s with an overlap of 5 s. Fig 5(A) shows the EEG recording of one patient and the whole process is divided into four parts: awake state, induction, unconsciousness and RoC state. Fig 5(B) shows the spectrogram of the EEG signal and it clearly presents the frequency changes in awake state, unconsciousness and RoC state. During the transition from awake state to unconsciousness, the EEG lost power in high frequencies, meanwhile, delta and alpha waves increased. The corresponding EEG measures at five scales were shown in Fig 5(C)–5(H).

**Fig 6:**
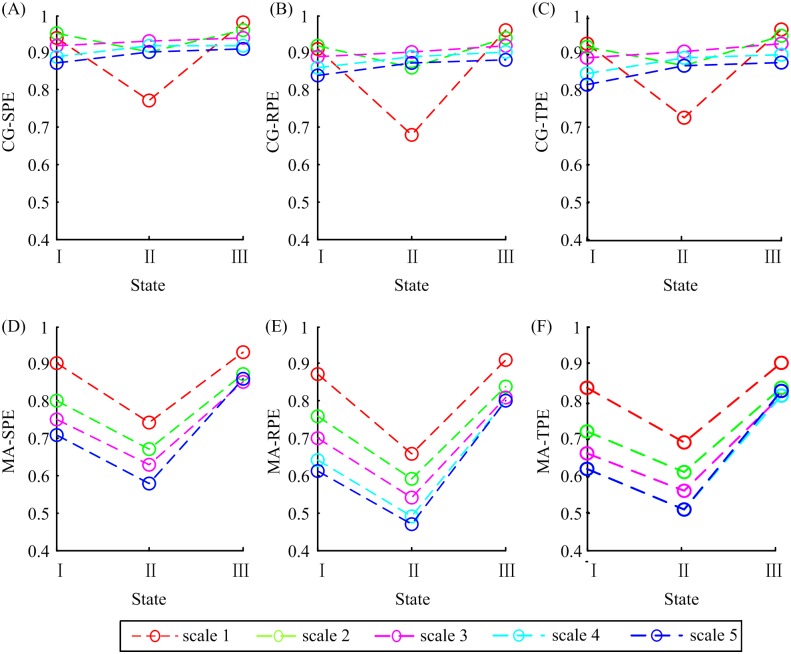
The median values of six MSPE indexes in awake state (I), unconsciousness (II) and RoC state (III). The circles symbolize the median values and different colors represent different decomposition scales.

To display the variation trend plainly, the median values of all the indexes from 20 patients in three states were plotted together for comparison in Fig 6. It is obvious that CG-SPE, CG-RPE and CG-TPE at scale 1 and 2 had the same trend, i.e. decrease in the anesthesia state and increase in the RoC state, while at scale 3, 4 and 5 the indexes rose in the anesthesia state and rose again in the RoC state, which violated the rule that PEs decrease in unconsciousness coupled with large amplitude, low-frequency waves. Therefore, the CG-based MSPEs lost efficacy at scale 3, 4 and 5 and this phenomenon may be related to shorten data length caused by CG decomposition method at high scales. In terms of MA-SPE, MA-RPE and MA-TPE, all scales had the same trends: drop in the anesthesia state and rise in the RoC state. Therefore, only CG-based MSPEs at *scale* = 1,2 and MA-based MSPEs can be used to track anesthesia signals. And only these methods were discussed in the following.

**Fig 7:**
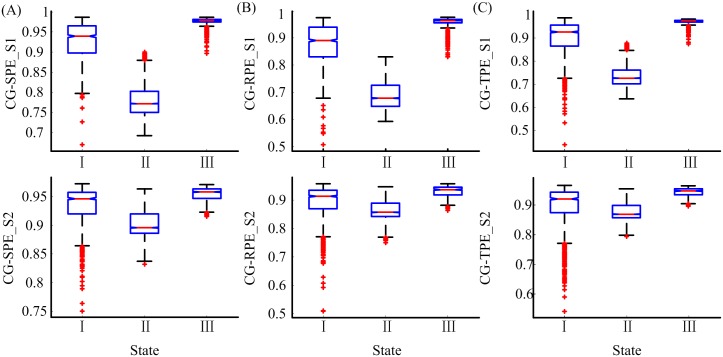
The statistical box plots of CG-SPE, CG-RPE and CG-TPE at scale 1 and 2 in awake state (I), unconsciousness (II) and RoC state (III). S1, S2 represent scale 1 and 2, respectively.

**Fig 8:**
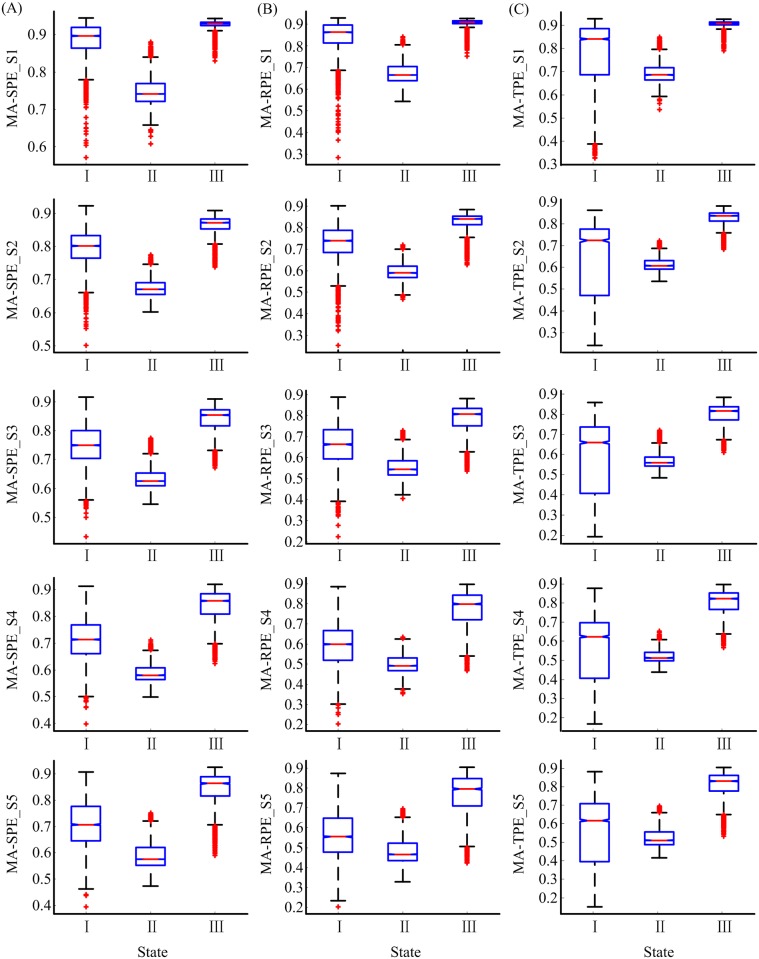
The statistical box plots of MA-SPE, MA-RPE and MA-TPE at scale 1–5 in awake state (I), unconsciousness (II) and RoC state (III). S1-S5 represent scale 1–5, respectively.

Six MSPE measures at scale 1 equal the corresponding PEs. The performance of the MSPEs was compared with PEs to discover the advantages of MSPEs. In order to compare the ability of the six measures in distinguishing different states, i.e., awake state, unconsciousness and RoC state, two box plots of CG-based and MA-based MSPE measures were given in Figs 7 and 8, respectively. The Kolmogorov-Smirnov test showed that all the indexes in different states were not normally distributed. The Kruskal-Wallis test and Multiple comparison test were adopted to estimate the significant difference among three states. All the significant differences of the six indexes were smaller than 0.001 (Kruskal-Wallis test and Multiple comparison test), and this illustrated that all the indexes can significantly distinguish awake state, unconsciousness and RoC state.

As can be seen from Figs 7 and 8, CG-based MSPEs at scale 2 had small variation between different states than at scale 1, while MA-based MSPEs had bigger variation range than CG group. The difference values between different states of MA-based MSPEs were summarized in Table 3. The sing-scale PEs had the biggest difference between unconsciousness and awake state (difference = −0.16, −0.19, −0.15), while the difference between RoC and unconsciousness state increased with decomposition scales. This verified that PEs can make a better distinction between unconsciousness and awake state, but MSPEs have better distinction ability between RoC and unconsciousness state. In terms of different PEs, SPE-based MSPEs had smallest range of variation. The differences between MA-SPE, MA-RPE, MA-TPE were negligible.

**Table 3:** The value difference of MA-based MSPEs between different states.

	Scale 1	Scale 2	Scale 3	Scale 4	Scale 5
MA-SPE	(-0.16, 0.19)^a^	(-0.13, 0.20)	(-0.12, 0.22)	(-0.13, 0.28)	(-0.13, 0.28)
MA-RPE	(-0.19, 0.25)	(-0.13, 0.25)	(-0.10, 0.27)	(-0.08, 0.31)	(-0.06, 0.33)
MA-TPE	(-0.15, 0.22)	(-0.11, 0.23)	(-0.10, 0.26)	(-0.11, 0.31)	(-0.11, 0.32)

^a^: (Index_*Unconsciousness*_−Index_*Awake*_, Index_*Recovery*_−Index_*Unconsciousness*_).

**Fig 9:**
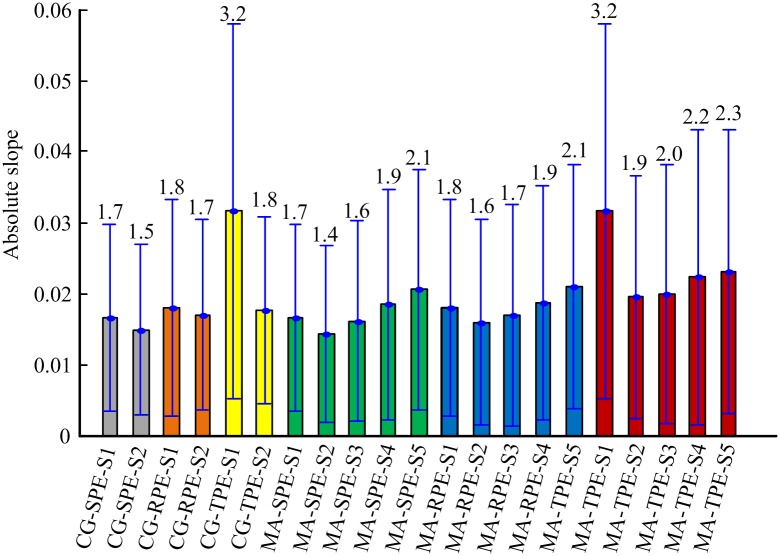
Statistical analysis of the absolute slope for six MSPE indexes at five scales. The numbers represent the mean values of absolute slope (*100) for each measure. The bar height indicates the mean value, and the lower and upper lines are the standard deviation of the measures.

Fig 9 shows the absolute slope of changes for six MSPEs during the transition from awake state to unconsciousness. As shown in the Fig 9, the absolute slopes of MSPEs at *scale* = 2,3 were smaller than PEs. MA-SPE and MA-RPE at *scale* = 4,5 had bigger absolute slope values than PEs, suggesting that MA-SPE and MA-RPE at *scale* = 4,5 could respond faster to the changes of DoA, especially at scale 5. Small differences were observed in tracking speed between MA-SPE and MA-RPE. MA-TPE had bigger absolute slopes than other indexes at the same decomposition level. Among the five scales of MA-TPE, single scale MS-TPE had the rapidest speed to the changes of DoA. The tracking speed increased with the increasing of decomposition scales for MA-TPE at scale 2–5.

**Fig 10:**
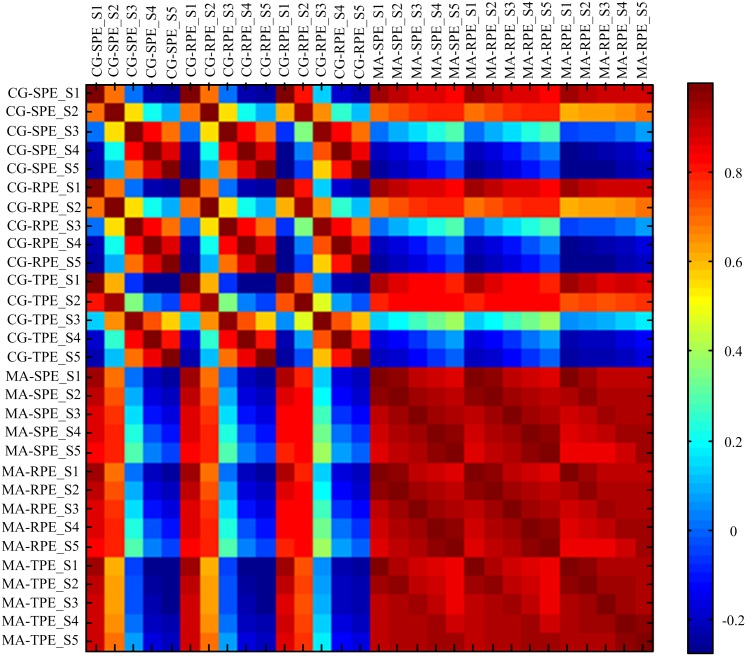
Correlation coefficient among six MSPE measures at five scales over 20 patients.

To further evaluate the relationship between the indexes, the correlation coefficients *R* were calculated and shown in Fig 10. Notably, the correlation coefficient between five scales of MA-SPE, MA-RPE and MA-TPE were all higher than 0.91, indicating that theses indexes correlated closely with each other. It can be seen from the figures that CG-based MSPEs at scale 1 and 2 had high correlation coefficient (R_min_ = 0.68) with other indexes, while CG-based MSPEs at scale 3–5 had low correlation coefficients with other indexes, which also indicates that they failed to track the features of anesthesia signals.

## 5. Conclusion and Discussions

Multiscale permutation entropy provides a new perspective in neural population analysis. In this study, six MSPE constructed by three kinds of permutation entropy measures (SPE, RPE and TPE) and two multiscale decomposition procedures CG and MA, were analyzed. In previous study, MSE measures were used for off-line EEG analysis and the results turned out that MSE measures could reflect the drug effect on the central nervous system [11]. In this paper, MSPE were designed for on-line DoA monitoring. Single-scale PEs were used as a test-bed to verify the significance of the new methods.

As we know, there are many other methods applied for DoA monitoring, such as: spectral edge frequency [44], median frequency [45–47], spectral entropy [48, 49], the bispectral index (BIS) [50–58], and the wavelet based index (WAV_CNS_) [59]. Among these methods, BIS is the most commonly used DoA index. However, previous studies found that the spectral feature indexes do not correlate with all anesthetic drugs in dose—response relation [60], and the BIS index is sensitive to artifacts, failed to regain its baseline value [61, 62]. Jin-Oh et al has verified that WAV_CNS_ exhibits linear time-invariant dynamics. On the other hand, methods based on nonlinear dynamics and information theory have been proposed to estimate DoA, including response entropy (RE) and state entropy (SE) [63], approximate entropy (AE) [64], sample entropy (SampEn) [16], fuzzy entropy (FuzzyEn) [65], permutation entropy (PE) [17, 66], Shannon wavelet entropy (SWE) [67], Hilbert-Huang spectral entropy (HHSE) [68], detrended fluctuation analysis (DFA) [69] and so on. In [18], the capability of 12 entropy indexes and DFA in DoA monitoring were compared and the result showed that three PE measures were superior than other entropies with less baseline variability, higher coefficient of determination and prediction probability [18]. This motivates us to explore the feasibility of PE-based MSE in DoA monitoring.

Except CG and MA procedures, there still exist other decomposition methods, such as wavelet transform (WT) and empirical mode decomposition (EMD). The intrinsic properties of these two procedures are time-frequency decomposition. They decompose the nonlinear neural oscillation signal into different frequency bands regularly and provide excellent performance in time-frequency domain analysis [70–72]. However, PE is based on the computation of symbolic dynamic of time series. Choosing WT and EMD as the decomposition method to construct MSE with PEs may induce lose effectiveness. Therefore, only CG and MA procedures were considered as decomposition methods in this study.

CG and MA procedures are typical morphology methods and both of them have advantages and disadvantages. The CG procedure reduces the length of a time series with the increase of scales. It is superior in long-term time series analysis but may yield an imprecise estimation of entropy in short-term time series [13]. In on-line signal analysis, since the length of the data gathered may be limited, CG-based MSPEs have poor performance in tracking characteristic features. MA method solves this problem, but brings computing redundancy.

In this paper, a TCNMM model was introduced to produce noise-free EEG and test the anti-noise ability of the six measures. Assuming the interactions among neural populations, and incorporating the bursting mode into the thalamic populations, the TCNMM model was constructed and we generated surrogate data of different anesthesia states fairly well by acting on the modulatory inputs. The power spectrum characters of different states were similar with the clinical EEG.

Through the simulated EEG and clinical EEG analysis, the performance of six MSPEs in anti-noise ability, tracking the strength changes of neural oscillations and distinguishing the different mental states were evaluated and concluded as follows:

1. In the aspect of anti-noise ability, CG-based MSPE measures had better anti-noise ability than MA-based MSPEs at the same decomposition level. In general, the increase of decomposition scale can enhance the robustness against white Gaussian noise, which verified that MSPEs outperformed PEs in this respect. Among SPE, RPE and TPE, RPE-based MSPEs had stronger noise resistance than others.
2. All the proposed MSPEs can significantly distinguish awake state, unconsciousness and RoC states during anesthesia. The influence of decomposition scales on discrimination ability was negligible.
3. Considering the opposite tracking trend of CG-based measures at scale = 3,4,5, CG-based MSPEs were not suitable for on-line anesthesia EEG analysis, but they had advantage in anti-noise in long-term data analysis. All the MA-based MSPE measures could track the strength changes of neural oscillations.
4. In terms of the tracking speed, the tracking speed increased with the increasing of decomposition scale from scale 2 to 5 for MA-based indexes. MA-SPE and MA-RPE at scale = 4,5 had faster response to DoA changes than PEs. MA-TPE has faster tracking speed than other MSPEs at the same decomposition level and MA-TPE at scale 1 has the biggest absolute slope among all the MSPEs.
5. Comparing MA-SPE, MA-RPE and MA-TPE, they have equal competence in distinguishing different anesthesia states, while MA-RPE has better anti-noise ability than the other two methods and MA-TPE can reveal the loss of consciousness faster.

In conclusion, we give an in-depth comparison of six MSPE measures and the results verified that MSPEs were superior to single-scale PEs with better robustness and faster tracking speed in online DoA monitoring. Synthesizing all the aspects, different MSPEs could be selected for clinical anesthesia EEG analysis according to their particular features.

However, the following issues should be addressed and need to be further explored. Firstly, the performance of different measures was only assessed with the TCNMM model and anesthesia EEG data sets. In consideration of the complexity of neural populations, the conclusion may not be suitable for all neurophysiological signals. Second, since MSPEs have different advantages in terms of different PEs and decomposition scales, in future study we could integrate into a composite index, which may better reflect the inner characteristics of the nonlinear signals. Further study is needed before incorporating the MSPE indexes into clinical DoA monitoring system.

## Supporting Information

S1 Fig The changes of SPE with different parameters in different anesthesia states. I-IV represent the combination of (m, τ) as (3, 1), (3, 2), (6, 1) and (6, 2), respectively. The red and green color represent the awake state and anesthesia state, respectively.
(TIF)

S2 Fig The changes of RPE and TPE with different parameters in different anesthesia states. (A): The changes of RPE with 0 < a < 1 and a > 1, a = 2 has the best discrimination ability. (B) The changes of TPE with 0 < *q* < 1 and *q* > 1, *q* = 0.1 has the best discrimination ability. The red and green color represent the awake state and anesthesia state, respectively.
(TIF)

S1 File The parameter selection of SPE, RPE and TPE; A thalamo-cortical neural mass model for the simulation of anesthesia. (DOCX)
