# Supplementary figures and images for: A Comparison of Multiscale Permutation Entropy Measures in On-Line Depth of Anesthesia Monitoring
Source: PLoS One. 2016 Oct 10;11(10):e0164104. doi: 10.1371/journal.pone.0164104 (PMC5056744; doi:10.1371/journal.pone.0164104)

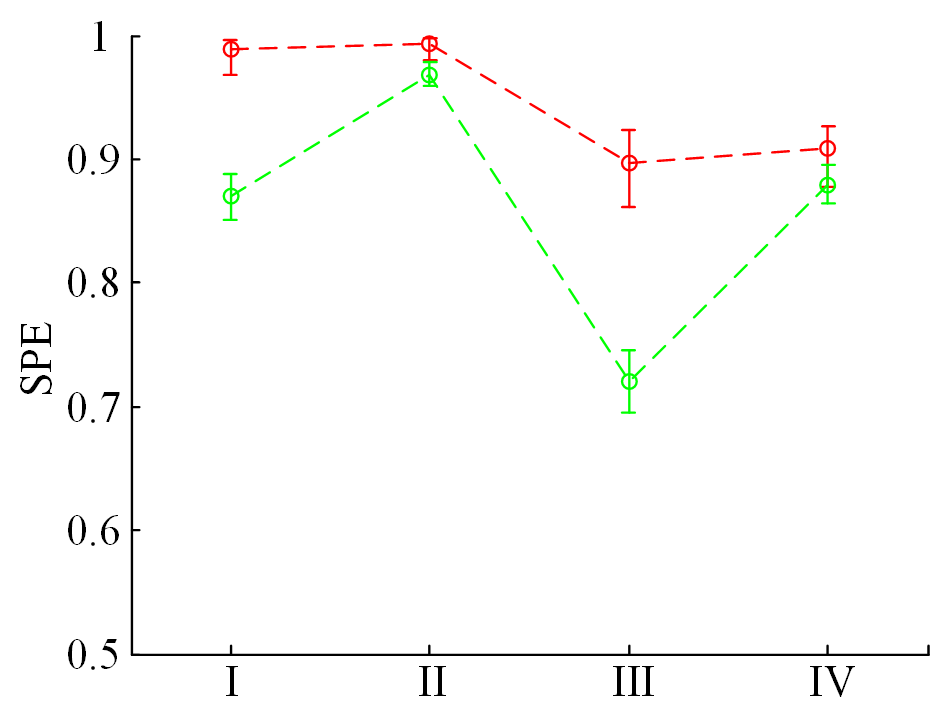

Supplement: S1 Fig — I-IV represent the combination of (m, τ) as (3, 1), (3, 2), (6, 1) and (6, 2), respectively. The red and green color represent the awake state and anesthesia state, respectively. (TIF) [file pone.0164104.s001.tif]

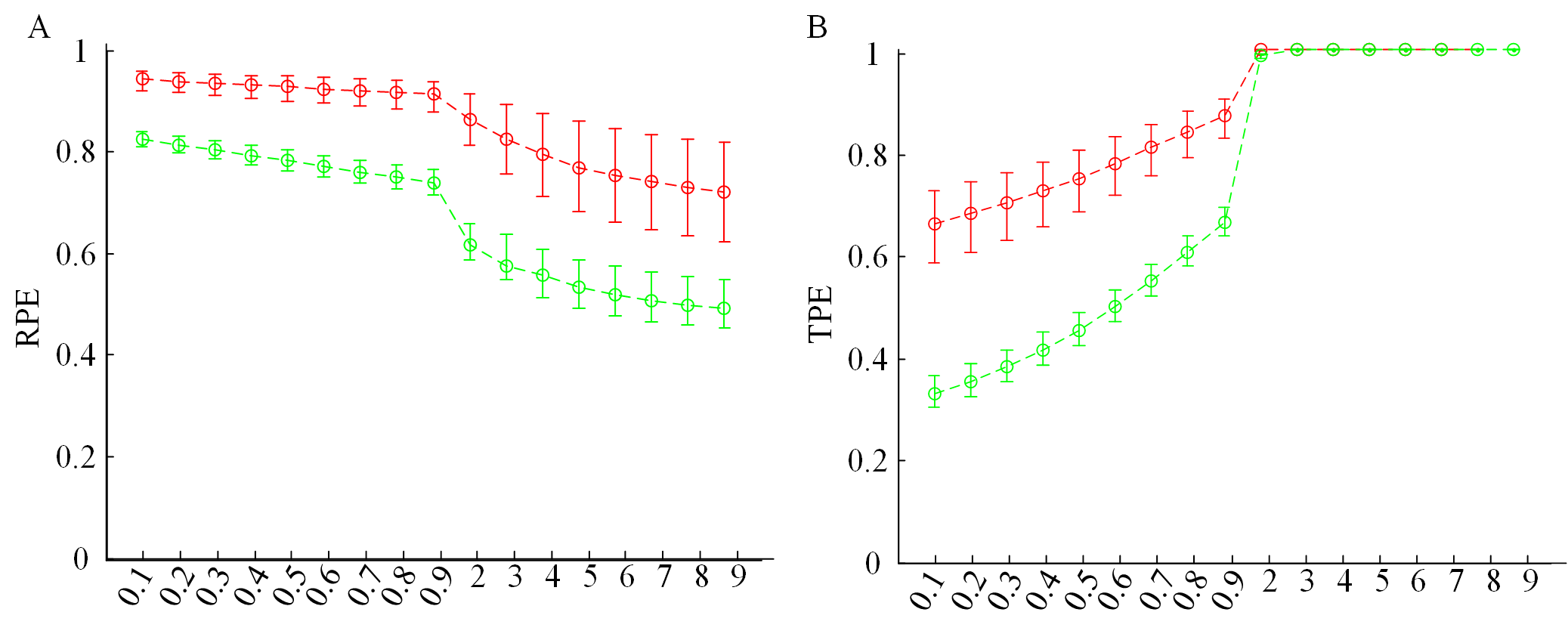

Supplement: S2 Fig — (A): The changes of RPE with 0 < a < 1 and a > 1, a = 2 has the best discrimination ability. (B) The changes of TPE with 0 < q < 1 and q > 1, q = 0.1 has the best discrimination ability. The red and green color represent the awake state and anesthesia state, respectively. (TIF) [file pone.0164104.s002.tif]
