# Supplementary material for: A Comparison of Multiscale Permutation Entropy Measures in On-Line Depth of Anesthesia Monitoring
Source: PLoS One. 2016 Oct 10;11(10):e0164104. doi: 10.1371/journal.pone.0164104 (PMC5056744; doi:10.1371/journal.pone.0164104)
Supplement: S1 File — (DOCX) [file pone.0164104.s003.docx]

**Supporting Information**

**The parameter selection of SPE, RPE and TPE**

The computation of SPE, RPE and TPE are based on several parameters. It is important to select appropriate parameters, since they have important influence on the results. The parameter selection was conducted with the EEG recordings described in section 4.1 and the performance of different parameters was evaluated on their ability of distinguishing awake and anesthesia states. The details of the parameter selection are discussed as follows. The red and green color represent the awake state and anesthesia state, respectively.

In previous study [1, 2], $m=6$, $\tau=1$ and $m=3$, $\tau=2$ are suggested for sevoflurane anesthesia and isoflurane anesthesia, respectively. Therefore, the embedded dimension m and τ can be combined as (3, 1), (3, 2), (6, 1) and (6, 2). S1 Fig showed the changes of SPE computed with the four groups and the results verified that m=6, τ=1 have the best performance in distinguishing different anesthesia states. As shown in S2(A) Fig, for the parameters of RPE, $0<a<1$ and $a>1$ were tested and the result showed that $a=6$ outperforms other values. As shown in S2(B) Fig, for the parameter of TPE, $0<q<1$ and $q>1$ were tested and the result shows that $q=0.1$ has the best performance. The select result of *q* is different with the previous study [3], due to that the EEG data in this paper was a result of the concerted action of multiple anesthetics.

**A thalamo-cortical neural mass model for the simulation of anesthesia**

The thalamo-cortical neural mass model is made up of thalamic populations and cortical populations. Average membrane potential $v_{i}(t)$ and average tonic firing rate $z_{i}(t)$ depict the main characters of each neuron. The neural population is described in the following.

$v_{i}\left( t \right)=\sum_{j} C_{ij}\cdot y_{j}\left( t-D_{ij} \right)+I_{ext,i}$ (1)

$r_{i}\left( t \right)=\frac{1}{1+e^{(v_{0,i}-v_{i}(t))/\sigma_{i}}}$ (2)

$z_{i}\left( t \right)=r_{i}(t)\cdot F_{i}$ (3)

$\ddot{y}_{i}\left( t \right)=G_{i}\cdot\omega_{i}\cdot z_{i}\left( t \right)-2\omega_{i}\cdot\dot{y}_{i}\left( t \right)-{\omega_{i}}^{2}\cdot y_{i}(t)$ (4)

$y_{i}\left( t \right)=z_{i}(t)\otimes h_{i}(t)$ (5)

$h_{i}\left( t \right)=G_{i}\cdot\omega_{i}\cdot t\cdot e^{-\omega_{i}\cdot t}\cdot u(t)$ (6)

The thalamic populations include two fire modes, i.e. tonic or with bursts. And the burst mode is described in the following:

$r_{B,i}\left( v_{i}\left( t \right) \right)=n(v_{i}(t))\cdot m(v_{i}(t))$ (7)

$\ddot{n}\left( v_{i}\left( t \right) \right)=\omega_{n1}\cdot\omega_{n2}\cdot n_{0}\left( v_{i}\left( t \right) \right)-\left( \omega_{n1}+\omega_{n2} \right)\cdot\dot{n}\left( v_{i}\left( t \right) \right)-\omega_{n1}\cdot\omega_{n2}\cdot n\left( v_{i}\left( t \right) \right)$ (8)

$n_{0}\left( v_{i}\left( t \right) \right)=\frac{1}{1+e^{(v_{0,n}-v_{i}(t))/\sigma_{n}}}$ (9)

$h_{n}\left( t \right)=\frac{\omega_{n1}\cdot\omega_{n2}}{\omega_{n1}+\omega_{n2}}(e^{-\omega_{n1}t}-e^{-\omega_{n2}t})\cdot u(t)$ (10)

$m\left( v_{i}\left( t \right) \right)=\frac{1}{1+e^{(v_{0,m}-v_{i}(t))/\sigma_{m}}}$ (11)

$z_{i}\left( t \right)=r_{B,i}\left( v_{i}\left( t \right) \right)\cdot F_{B}+(1-r_{B,i}\left( v_{i}\left( t \right) \right))\cdot r_{i}(t)\cdot F_{i}$ (12)

Unlike other neurons in the model, the firing rate of $\mathrm{GABA}_{B}$ receptors is described as a nonlinear, sigmoidal activation function.

$z_{{GABA}_{B}}\left( t \right)=z_{R}(t)\frac{1}{1+e^{(z_{0,{GABA}_{B}}-z_{R}(t))/\sigma_{{GABA}_{B}}}}$ (13)

In terms of $\mathrm{GABA}_{A}$ and $\mathrm{GABA}_{B}$, the post-synaptic inhibition is described as:

$y_{R}\left( t \right)=z_{R}\left( t \right)\otimes h_{{GABA}_{A}}\left( t \right)+z_{{GABA}_{B}}\left( t \right)\otimes h_{{GABA}_{B}}\left( t \right)$ (14)

The detail of all the values of parameters in the equations can be found in.

To generate the anesthesia EEG by the thalamo-cortical neural mass model, three inputs reaching the TCR ($I_{M,T}$), the TRN ($I_{M,R}$) and the pyramidal ($I_{M,P}$) populations are modulated to switch between awake state, unconsciousness and RoC state. The values of the three inputs are fixed when the power spectrum of produced signal meets the power spectrum of real EEG in different states.

1. Li X, Cui S, Voss LJ. Using permutation entropy to measure the electroencephalographic effects of sevoflurane. Anesthesiology. 2008;109(3):448.

2. Li D, Liang Z, Wang Y, Hagihira S, Sleigh JW, Li X. Parameter selection in permutation entropy for an electroencephalographic measure of isoflurane anesthetic drug effect. J Clin Monit Comput. 2012. Epub 2012/12/25. doi: 10.1007/s10877-012-9419-0. PubMed PMID: 23264067.

3. Liang Z, Wang Y, Sun X, Li D, Voss LJ, Sleigh JW, et al. EEG entropy measures in anesthesia. Frontiers in Computational Neuroscience. 2015;9:16.
